# Oleocanthal Ameliorates Metabolic and Behavioral Phenotypes in a Mouse Model of Alzheimer’s Disease

**DOI:** 10.3390/molecules28145592

**Published:** 2023-07-23

**Authors:** Euitaek Yang, Junwei Wang, Lauren N. Woodie, Michael W. Greene, Amal Kaddoumi

**Affiliations:** 1 Department of Drug Discovery and Development, Harrison College of Pharmacy, Auburn University, 720 S Donahue Dr., Auburn, AL 36849, USA; ezy0014@auburn.edu (E.Y.); jzw0164@auburn.edu (J.W.); 2 Department of Nutrition, College of Human Sciences, Auburn University, Auburn, AL 36849, USA; lauren.woodie@pennmedicine.upenn.edu (L.N.W.); mwg0006@auburn.edu (M.W.G.)

**Keywords:** Alzheimer’s disease, blood–brain barrier, metabolic phenotype, sleep behavior, Promethion cages, oleocanthal

## Abstract

Aging is a major risk factor for Alzheimer’s disease (AD). AD mouse models are frequently used to assess pathology, behavior, and memory in AD research. While the pathological characteristics of AD are well established, our understanding of the changes in the metabolic phenotypes with age and pathology is limited. In this work, we used the Promethion cage systems^®^ to monitor changes in physiological metabolic and behavioral parameters with age and pathology in wild-type and 5xFAD mouse models. Then, we assessed whether these parameters could be altered by treatment with oleocanthal, a phenolic compound with neuroprotective properties. Findings demonstrated metabolic parameters such as body weight, food and water intake, energy expenditure, dehydration, and respiratory exchange rate, and the behavioral parameters of sleep patterns and anxiety-like behavior are altered by age and pathology. However, the effect of pathology on these parameters was significantly greater than normal aging, which could be linked to amyloid-β deposition and blood–brain barrier (BBB) disruption. In addition, and for the first time, our findings suggest an inverse correlation between sleep hours and BBB breakdown. Treatment with oleocanthal improved the assessed parameters and reduced anxiety-like behavior symptoms and sleep disturbances. In conclusion, aging and AD are associated with metabolism and behavior changes, with the changes being greater with the latter, which were rectified by oleocanthal. In addition, our findings suggest that monitoring changes in metabolic and behavioral phenotypes could provide a valuable tool to assess disease severity and treatment efficacy in AD mouse models.

## 1. Introduction

Alzheimer’s disease (AD) is a neurodegenerative disorder causing dementia. Age is considered a major risk factor for developing AD. Aging alters many physiological processes in the human body and metabolic and behavioral phenotypes [1]. Metabolic and behavioral phenotyping is used to assess alterations in physiological metabolism and behavior that are affected by numerous factors, including diet, lifestyle, disease conditions, and other environmental factors [2]. Examples of metabolic and behavioral phenotypes that are affected by age and disease include food and water intake, activity, movement, energy expenditure, respiratory exchange, hydration, and sleep patterns [3].

While the pathological characteristics of AD are well established, our understanding of the changes in the metabolic phenotypes with age and pathology continues to be limited. Several studies have reported appetite loss in persons with mild cognitive impairment (MCI) and dementia [4]. Decreased meal consumption could result in malnutrition, dehydration, failing of body homeostasis, weakening of immunity, and reduction in cognitive function [5]. In AD patients, a range of factors can affect appetite, including fastidiousness in eating and the inability to feel hungry because of brain atrophy, disturbance of eating behavior, loss of the ability to use eating utensils, and decrease in swallowing function [6]. A change in food intake could affect other metabolic parameters, such as activity and energy consumption [7]. In addition, AD patients experience sleep disturbances that may precede the other clinical signs of AD [8]. Sleep disorders may affect the circadian rhythm, which has been linked to fluctuations in amyloid-β (Aβ) levels in interstitial brain fluid (ISF) and cerebrospinal fluid (CSF) [9,10,11,12]. In the adult brain, the clearance of Aβ during sleep is two-fold faster than during wakefulness [13], and brain Aβ accumulation induces excessive daytime sleepiness [14]. In humans, monitoring the sleep–wake cycle in individuals with AD aged 45–75 years has shown a decreased sleep efficiency and increased nap frequency in individuals with Aβ deposition compared with individuals of the same age but without Aβ deposition as determined by CSF-Aβ_42_ level [15]. Thus, AD could affect the sleep pattern, and at the same time, sleep disturbances might contribute to AD progression [8]. Besides sleep disturbances, anxiety is one of the neuropsychiatric symptoms of AD [16]. Anxiety has also been considered a risk factor for AD, especially in midlife [17]. AD patients exhibit anxiety at the MCI stage, which is associated with an increased likelihood of dementia [18].

AD research frequently uses mouse models to understand the pathology and behavior and test potential treatments. Studies investigating mitochondrial respiratory function, circadian-related proteins, eye movement, and electroencephalography are often used to test the pathology effect on metabolism and circadian rhythms in mice [19,20,21,22,23]. Open field, elevated plus-maze, and light/dark tests have been used for anxiety-like behavior [18]. These methodologies are performed in brain tissues or require mouse restraining or training, which could introduce a stress factor that could confound the results.

Promethion cage systems^®^ are a valuable tool for metabolic and behavioral phenotyping that simultaneously monitors metabolic data with behavioral events under natural conditions without introducing a stress factor to the animals. Examples of metabolic and behavior parameters that these systems could obtain include body weight, food and water intake, movement, energy expenditure, dehydration level, and respiratory exchange. Based on the mouse behavior and movement monitored by sensors in the cages, sleep pattern and anxiety-like behavior could be determined (Table 1).

In this work, we aimed to utilize the Promethion cage system to compare differences in metabolic and behavioral parameters as a function of age (4 vs. 9 months) and pathology in wild-type (WT) and 5xFAD as a mouse model of AD and then to evaluate whether the assessed metabolic and behavioral phenotypes respond to oleocanthal (OC) as a treatment for AD. OC is a naturally occurring phenolic secoiridoid isolated from extra-virgin olive oil (EVOO), which possesses anti-inflammatory effects similar to the nonsteroidal anti-inflammatory drug ibuprofen [24]. Studies from our laboratory have reported the beneficial effect of OC in AD mouse models where OC treatment reduced brain Aβ levels, improved the blood–brain barrier (BBB) function, reduced neuroinflammation, enhanced autophagy, and improved memory function [25,26,27,28].

We demonstrate here that age and pathology are associated with sleep disturbances, altered energy expenditure, activity rate, and moved distances associated with anxiety-like behavior. Between the two age groups, 9-month-old mice demonstrated greater differences in the monitored parameters than the younger group. Furthermore, our results indicate that 5xFAD mice treated with OC, 10 mg/kg, improved several of the assessed parameters to levels similar to or approaching those of the WT mice. Our findings suggest that metabolic and behavioral phenotypes are altered with age and pathology and support their use to monitor disease progression, severity, and treatment response as an additional approach to conventional currently used approaches.

## 2. Results

### 2.1. Effect of Age on the Phenotypic Parameters in WT and 5xFAD Mice

**Scheme 1:**
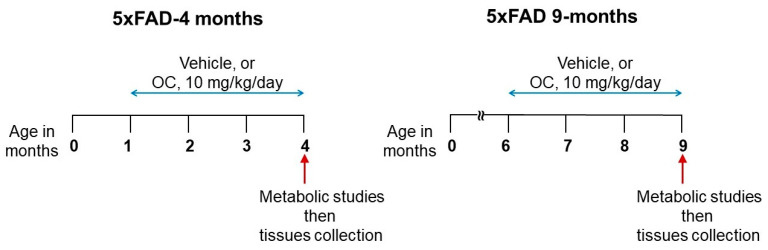
A schematic diagram of the experimental design. Four- and nine-month-old. WT mice received only the vehicle.

A schematic diagram of the animals’ experimental design is presented in Scheme 1,. The metabolic phenotype parameters were measured over a 24 h period starting at zeitgeber time (ZT) 0 (6 am). ZT0 represents lights on, and ZT12 represents lights off. All assessed parameters are summarized in Table 1.

Figures S1 and S2 (Supplementary data) show the effect of age (4 vs. 9 months) on assessed parameters in WT and 5xFAD mice, respectively, over the 24 h diurnal rhythm time. Tables S1 and S2 list the significance of the difference of each parameter at each time point for WT and 5xFAD mice, respectively. As shown in Figure S1 and Table S1, as expected, a gain in body weight was observed with normal aging. In addition, time points comparison of the parameters demonstrated a significant reduction in mean energy expenditure (EE), the mean rate of carbon dioxide emission (VCO_2_), the mean rate of oxygen consumption (VO_2_), respiratory exchange ratio (RER), and mean rate of water vapor loss (VH_2_O) in WT-9-month-old (WT-9m) mice compared to WT-4-month-old (WT-4m) mice across multiple time points in the ZT. On the other hand, in 5xFAD mice, except for the VH_2_O (at daytime and nighttime), distance traveled (meters), and cumulative distance traveled (mainly nighttime) showed a significant increase with aging. However, monitored changes of other assessed parameters over time between 0–24 h did not show significant alteration between 4-(5xFAD-4m) and 9-(5xFAD-9m) month-old mice (Figure S2 and Table S2).

**Figure 1:**
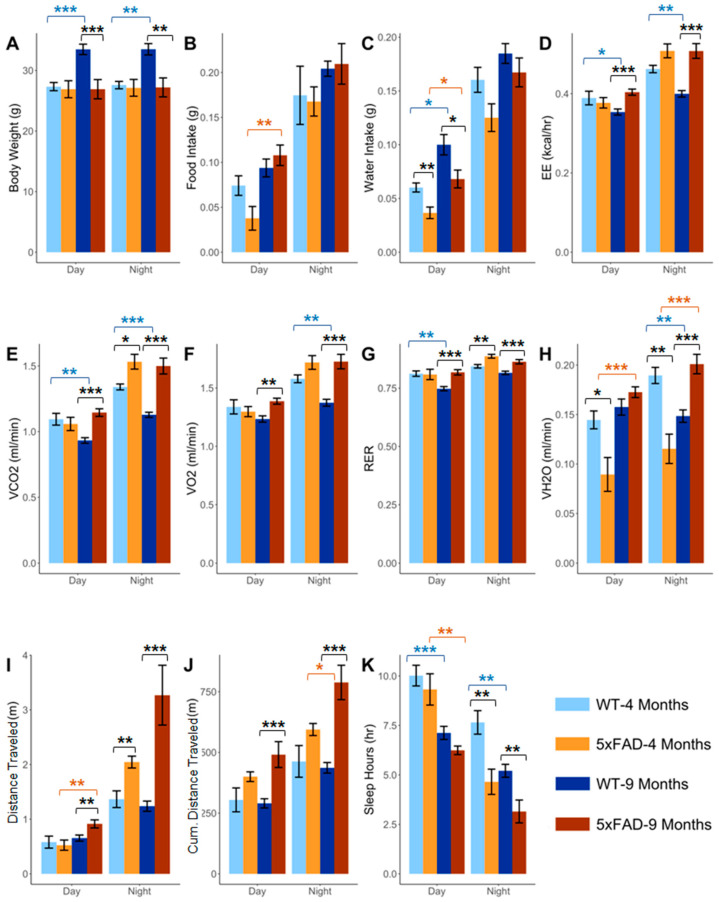
The effect of aging and pathology on the metabolic parameters (**A**) Body weight (g), (**B**) Food intake (g), (**C**) Water intake (g), (**D**) EE (kcal/h), (**E**) VCO_2_ (mL/min), **(F)** VO_2_ (mL/min), (**G**) RER, (**H**) VH_2_O (mL/min), (**I**) Distance traveled (m), (**J**) Cumulative (Cum.) distance traveled (m), and (**K**) Sleep hours (h) in WT and 5xFAD mice at daytime and nighttime. The statistical significance is color-coded with blue stars indicating the difference in WT with age, orange stars indicating the difference in 5xFAD mice with age, and black stars indicating the difference between WT and 5xFAD mice. Data are presented as mean ± SEM for *n* = 10 mice/group. * *p* < 0.05, ** *p* < 0.01, and *** *p* < 0.001.

Figure 1 demonstrates the 12 h circadian (day/night) data for the effect of age and pathology on the parameters at day (average of ZT0-11) and night (average of ZT12-23) times. As shown in Figure 1A, in WT and 5xFAD mice, 9-month-old mice have higher body weight than the 4-month-old mice. WT-9m mice exhibited a significant body weight gain by 22% more than WT-4m, an effect not observed in 5xFAD mice where 4- and 9-month-old mice exhibited similar body weight (about 27 g). During the daytime, no significant difference was observed between WT-4m and WT-9m mice in food intake (Figure 1B), VH_2_0 (Figure 1H), and distance traveled (total and accumulative distance; Figure 1I,J), while a significant increase in water intake was observed (Figure 1C). However, the data demonstrated a significant reduction in EE, VCO_2_, VCO_2_, and RER parameters (Figure 1D–G) in WT-9m compared to WT-4m mice. At nighttime, the older WT mice significantly reduced EE, VCO_2_, VO_2_, and VH_2_O compared to the young mice (Figure 1D–F,H). In 5xFAD mice, with age, at daytime, a significant increase in food and water intake, VH_2_O, and cumulative distance traveled were observed (Figure 1B,C,H,I). At nighttime, 5xFAD-9m mice demonstrated a significant increase in VH_2_O and cumulative distance traveled compared to 5xFAD-4m mice (Figure 1H,J). These results suggest that aging in WT mice is associated with reduced metabolic activity, while 5xFAD demonstrated either a no change or an increase in metabolic activity with aging.

### 2.2. Effect of Pathology on the Phenotypic Parameters at 4 and 9 Months in 5xFAD

Figures S3 and S4 demonstrate the effect of pathology on assessed parameters over time in WT and 5xFAD mice at 4 and 9 months of age, respectively. At 4 months, 5xFAD-4m mice demonstrated a significant reduction in the VH_2_O parameter during the 24 h (Figure S3H). While there was a significant increase or a trend for an increase in the metabolic parameters at a few time points between ZT12-23 h (Figure S3, Table S3), which was better demonstrated in the daytime and nighttime data shown in Figure 1. On the other hand, compared to WT mice, at the age of 9 months, 5xFAD mice demonstrated a significant increase in all assessed metabolic parameters almost at all time points between ZT0-23 (Figure S4, Table S4), suggesting a significant pathology effect in older mice.

Regarding the effect of pathology on body weight, there was no significant difference between WT-4m and 5xFAD-4m mice. However, 5xFAD-9m mice demonstrated a significantly lower body weight than WT-9m mice by 20%; 5xFAD-9m mice exhibited a body weight similar to the 4-month-old WT and 5xFAD mice (27 g), suggesting the impact of advanced pathology on body weight (Figure 1A). Within the daytime (Figure 1), while 5xFAD-4m mice demonstrated lower food intake than WT-4m mice, this difference was insignificant (Figure 1B). Water intake, however, was significantly lower in 5xFAD-4m than in WT-4m mice (Figure 1C). All other parameters were comparable to WT mice except for the VH_2_O parameter, where 5xFAD-4m mice exhibited a significantly lower rate of water loss than WT-4m mice (Figure 1H). On the other hand, at 9 months of age, 5xFAD demonstrated a significant reduction in water intake and a significant increase in the metabolic parameters such as EE, VCO_2_, VO_2_, RER, distance traveled, and cumulative distance traveled (Figure 1D–H,I,J). At nighttime, however, 5xFAD-4m mice showed a significant increase in VCO_2_, RER, and distance traveled, associated with a significant reduction in VH_2_O compared to WT-4m mice. Like the changes at 4 months, 9-month-old 5xFAD mice maintained a significant increase in the metabolic parameters EE, VCO_2_, VO_2_, RER, VH_2_O, distance traveled, and cumulative distance traveled (Figure 1D–J). Changes in the metabolic parameters with pathology indicate an anxiety-like behavior characterized by restlessness (increased movement and moving distances), sweating (increased water loss, VH_2_O), sleep disturbances, and rapid breathing (VO_2_, VCo_2_, and RER), which collectively suggest that compared to WT mice, 5xFAD mice exhibit an anxiety-like behavior.

### 2.3. Effect of Age and Pathology on Sleep Pattern in WT and 5xFAD Mice

The sleep parameter was assessed as the proportion of cycles when a mouse sleeps, that is, when a mouse stays quiet for more than 40 s. As shown in Figure 1K, consistent with the circadian biology of mice, sleeping time during the daytime was observed to be longer than the nighttime in both mouse models. Sleeping behavior was also influenced by age and pathology. At day and night times, 9-month-old WT and 5xFAD mice demonstrated a significantly lower sleeping time by approximately 35% compared to the 4-month-old mice. For the effect of pathology on sleeping duration, during the daytime, while 5xFAD demonstrated a reduction trend, the effect was not significant between the two mouse models at both ages; during the nighttime, however, younger and older 5xFAD mice slept about 40% fewer hours than the WT mice.

### 2.4. Effect of Age and Pathology on BBB Function

**Figure 2:**
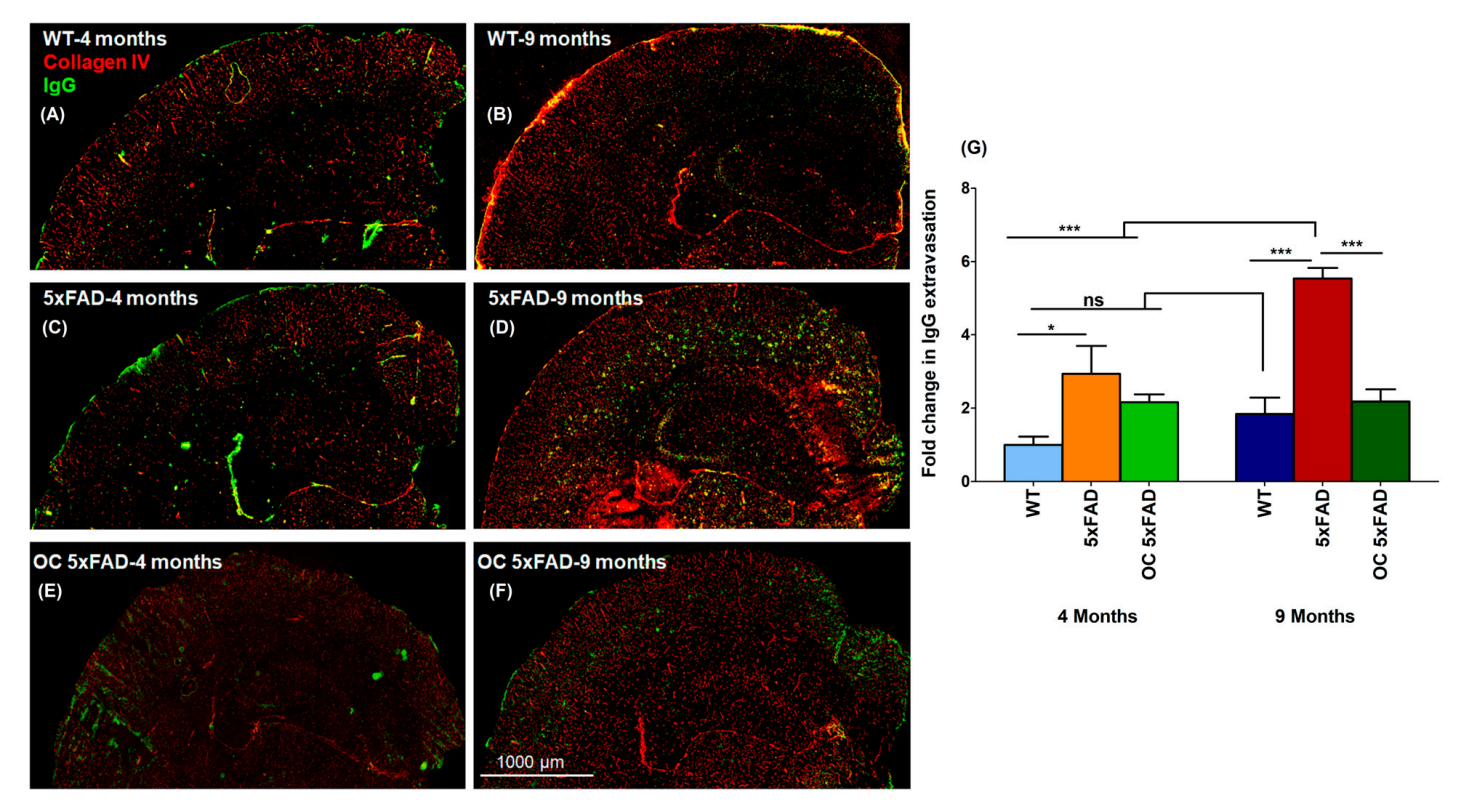
Representative brain sections stained with anti-mouse IgG antibody to detect IgG extravasation (green) and anti-collagen antibody (red) in mouse brain (**A**) WT-4-month-old, (**B**) WT-9-month-old, (**C**) 5xFAD-4-month-old, (**D**) 5xFAD-9-month-old, (**E**) 5xFAD-4-month-old treated with 10 mg/kg OC daily for 3 months, and (**F**) 5xFAD-9-month-old treated with 10 mg/kg OC daily for 3 months. (**G**) IgG optical density in mice brains was quantified for IgG extravasation. Data are presented as mean + SEM for *n* = 5 mice/group. ns for not significant, * *p* < 0.05, and *** *p* < 0.001 vs. WT-4 months WT mice. Scale bar, 1000 μm.

In AD mouse models, extravasation of large molecular size proteins, such as IgG, is commonly observed [28,29]. Thus, IgG extravasation in mouse brains was assessed using immunofluorescence to evaluate the effect of age and pathology on BBB function. As shown in Figure 2, a significant IgG extravasation was observed in 5xFAD-9m mice. For the effect of age, while WT-9m demonstrated a trend of increase in IgG extravasation compared to the WT-4m mice, the effect was insignificant. In 5xFAD-9m mice, a significantly higher IgG extravasation was observed by 1.9-fold compared to 5xFAD-4m mice. For the effect of pathology, at both ages, 5xFAD mice demonstrated a significantly higher IgG, i.e., three-fold higher, extravasation than WT mice.

### 2.5. Effect of Age and Pathology on Plasma and Brain Aβ Levels in WT and 5xFAD Mice

**Figure 3:**
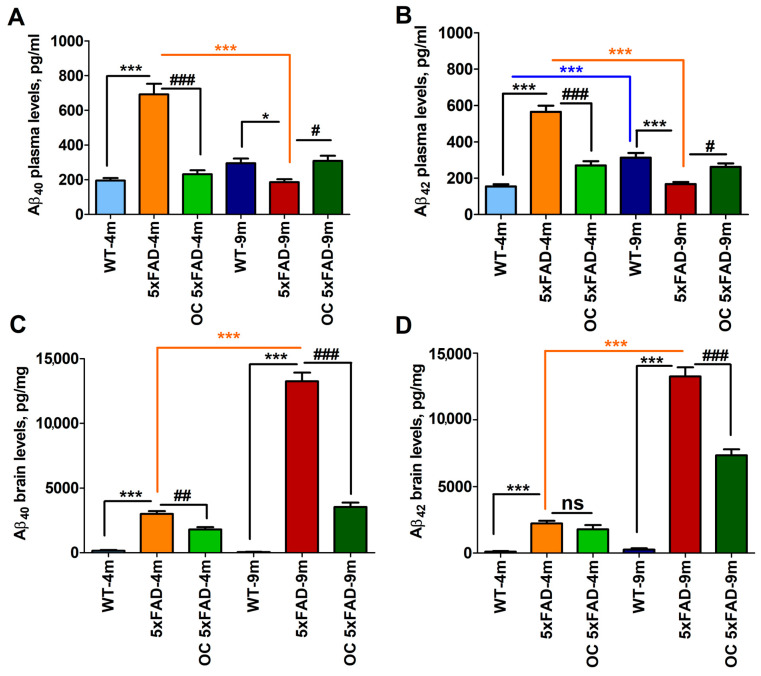
The effect of age, pathology, and 10 mg/kg OC treatment on levels of soluble plasma Aβ_40_ (**A**) and Aβ_42_ (**B**) and brain Aβ_40_ (**C**) and Aβ_42_ (**D**) as determined using ELISA in WT and 5xFAD mice. The statistical significance is color-coded with blue stars indicating the difference in WT with age, orange stars indicating the difference in 5xFAD mice with age, and black stars indicating the difference between WT and 5xFAD mice. Data are presented as mean ± SEM (*n* = 10/group). ns for not significant, * *p* < 0.05, *** *p* < 0.001, # *p* < 0.05, ## *p* < 0.01, and ### *p* < 0.001.

Plasma and brain Aβ_40_ and Aβ_42_ levels were analyzed using ELISA. As shown in Figure 3A, for Aβ_40_, there was a trend of increased plasma levels in 9-month-old compared to 4-month-old WT mice, but this increase was insignificant. On the other hand, for plasma Aβ_42_, 9-month-old WT demonstrated two-fold higher levels than the 4-month-old mice (Figure 3B). Interestingly, 5xFAD-9m showed significantly lower Aβ_40_ and Aβ_42_ plasma levels by 74 and 70%, respectively, compared to the 5xFAD-4m mice. For the effect of pathology, at 4 months of age, the plasma levels of Aβ_40_ and Aβ_42_ in 5xFAD mice are 3- and 2.1-fold higher than in WT-4-month-old mice; as the mice aged, the plasma levels of Aβ_40_ and Aβ_42_ in 5xFAD-9-month-old mice were significantly lower than WT-9-month-old mice by 36% and 47%, respectively.

In the brain, as shown in Figure 3C,D, Aβ_40_ and Aβ_42_ levels in the 9-month-old 5xFAD were significantly higher than 4-month-old 5xFAD by 4.4- and 6.0-fold, respectively; at both ages, brain Aβ_40_ and Aβ_42_ levels in 5xFAD mice were significantly higher than the WT mice, which showed negligible levels of Aβ_40_ and Aβ_42_ (Figure 3C,D).

### 2.6. Brain Soluble Aβ, IgG Extravasation, and Sleep Correlation

**Figure 4:**
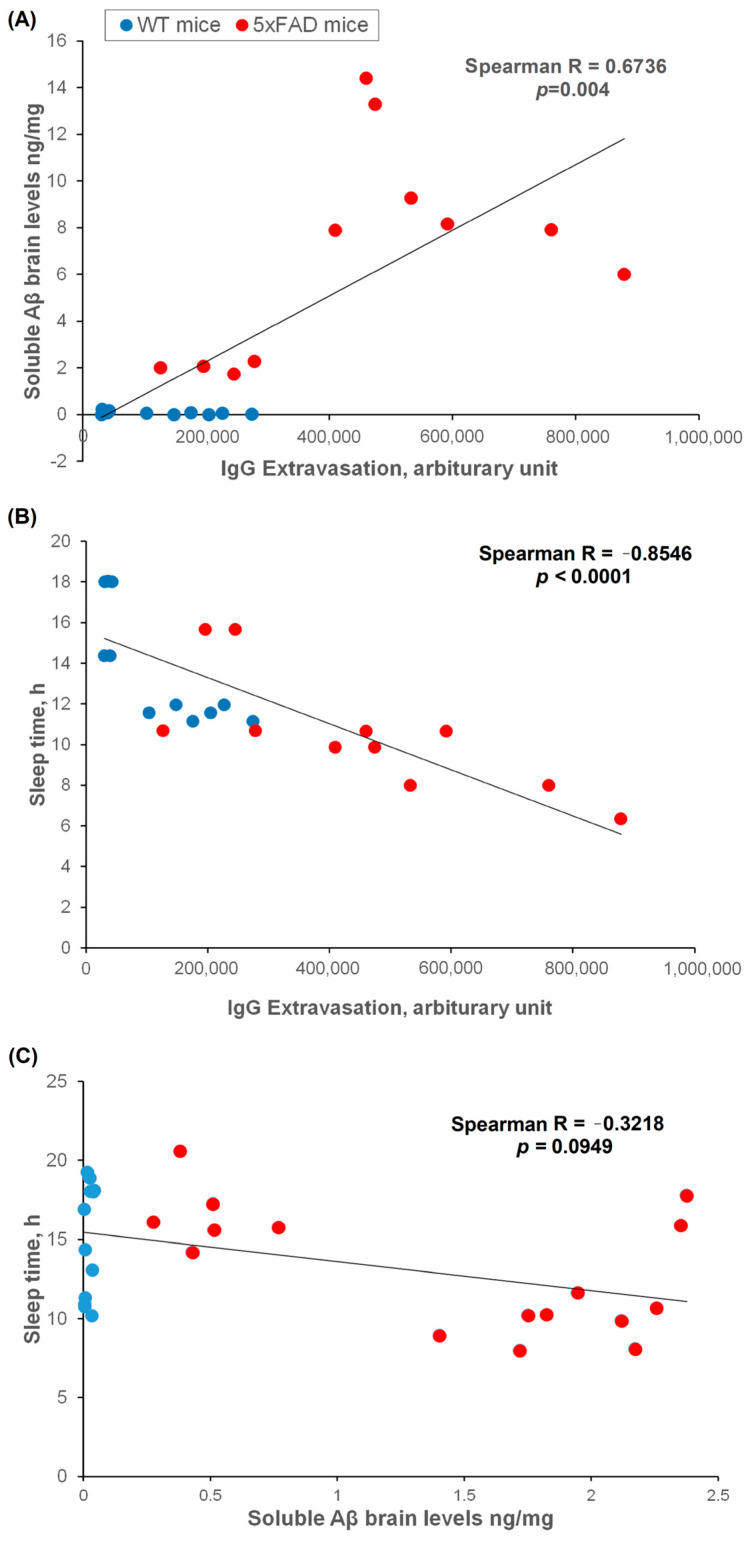
Correlation analysis between IgG extravasation and soluble brain Aβ levels (**A**) and sleep time (**B**), and between soluble brain Aβ and sleep time (**C**). For A and B, *n* = 11 mice/group were used; for C, *n* = 16 mice/group were used.

We performed the Spearman correlation analysis to clarify the relationship between brain soluble Aβ, IgG extravasation, and total sleep hours and whether the correlation is significant. As shown in Figure 4, and as expected, a positive correlation with Spearman R = 0.6736 (*p* = 0.004) between brain soluble Aβ levels and IgG extravasation was observed, supporting Aβ contribution to BBB breakdown. In addition, an inverse correlation between total sleep time and IgG extravasation was also observed (Spearman R = −0.8546; *p* < 0.0001); however, a weak correlation between brain soluble Aβ and sleep time was observed with Spearman R = −0.3218 (*p* = 0.0949).

### 2.7. Effect of OC Treatment on Metabolic Phenotypes, Aβ, and Related Pathology

**Figure 5:**
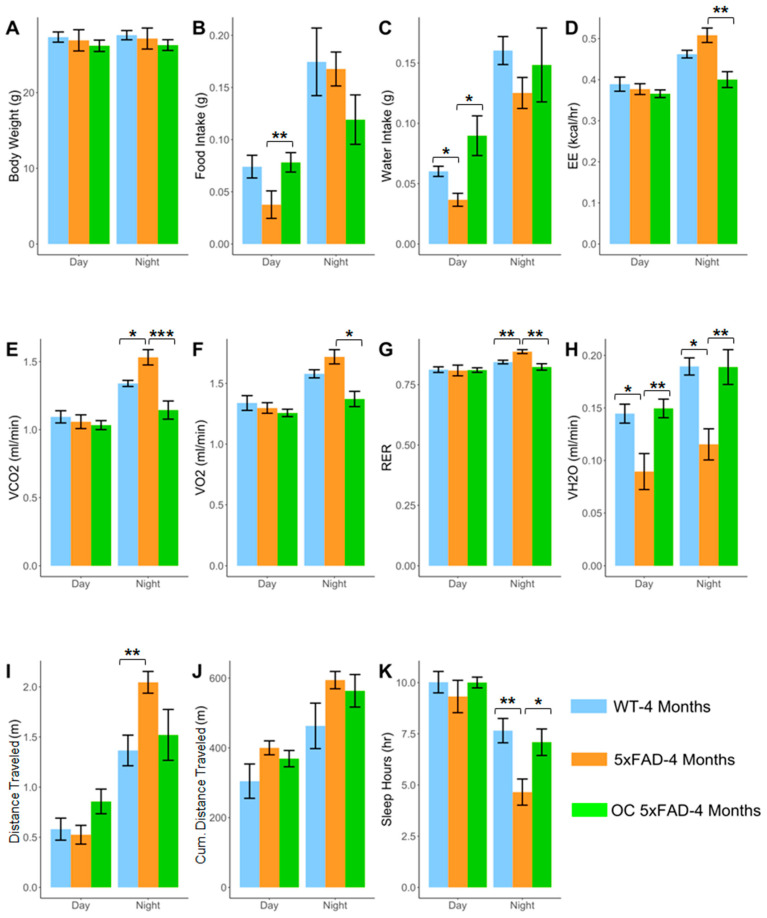
The effect of OC (10 mg/kg; PO) on the metabolic parameters (**A**) Body weight (g), (**B**) Food intake (g), (**C**) Water intake (g), (**D**) EE (kcal/h), (**E**) VCO_2_ (mL/min), (**F**) VO_2_ (mL/min), (**G**) RER, (**H**) VH_2_O (mL/min), (**I**) Distance traveled (m), (**J**) Cumulative (Cum.) distance traveled (m), and (**K**) Sleep hours (h) in 4-month-old WT and 5xFAD mice at daytime and nighttime. Data are presented as mean ± SEM for *n* = 10 mice/group. * *p* < 0.05, ** *p* < 0.01, and *** *p* < 0.001.

**Figure 6:**
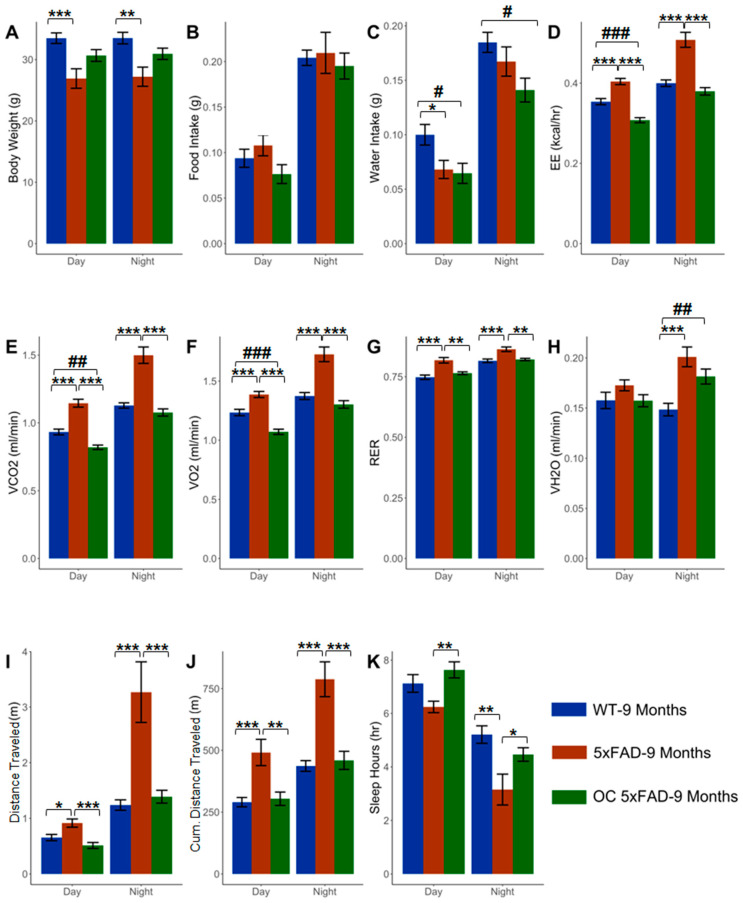
The effect of OC (10 mg/kg; PO) on the metabolic parameters (**A**) Body weight (g), (**B**) Food intake (g), (**C**) Water intake (g), (**D**) EE (kcal/h), (**E**) VCO_2_ (mL/min), (**F**) VO_2_ (mL/min), (**G**) RER, (**H**) VH_2_O (mL/min), (**I**) Distance traveled (m), (**J**) Cumulative (Cum.) distance traveled (m), and (**K**) Sleep hours (h) in 9-month-old WT and 5xFAD mice at daytime and nighttime. Data are presented as mean ± SEM for *n* = 10 mice/group. * *p* < 0.05, ** *p* < 0.01, *** *p* < 0.001, # *p* < 0.05, ## *p* < 0.01, and ### *p* < 0.001.

In young 5xFAD-4m mice (Figure 5), the daily treatment with OC (10 mg/kg) by oral gavage for 3 months significantly altered metabolic parameters. OC significantly increased food and water intake during the daytime compared to vehicle-treated 5xFAD mice without altering the body weight (Figure 5A–C). In addition, compared to vehicle-treated 5xFAD mice, mainly at nighttime, OC significantly reduced EE, VCO_2_, VO_2_, and RER to levels comparable to WT mice (Figure 5D–G). This effect was associated with an increased rate of water loss (VH_2_O) during the daytime and nighttime to levels similar to WT-4 m (Figure 5H) and significantly reduced movement and increased sleep time at nighttime to levels comparable to WT-4m mice (Figure 5I,K). Interestingly, the effect of OC treatment on the older 5xFAD mice (9 months) was more prominent. While OC increased 5xFAD-9m mice body weights by 4.0 g, the effect did not reach a significant effect compared to 5xFAD vehicle-treated mice; however, they were comparable to WT mice (Figure 6A). In addition, OC treatment significantly increased daytime and nighttime sleep hours by 1.4 and 1.2 h, respectively, approaching WT-9m mice (Figure 6K); OC treatment significantly reduced EE, VCO_2_, VO_2_, RER, distance traveled, and accumulative distance traveled almost to levels similar to those obtained with WT-9-month-old mice (Figure 6D–J), without altering food and water intake (Figure 5B,C). Changes in metabolic parameters suggest OC ameliorated parameters related to anxiety-linked behavior and sleep.

For OC effect on Aβ plasma and brain levels, as shown in Figure 3, OC-treated 5xFAD-4m mice demonstrated a significantly reduced plasma Aβ_40_ and Aβ_42_ by 67% and 52% approaching WT-4m levels, while increased their levels in 5xFAD-9m by 1.66- and 1.56-fold approaching the WT-9m levels. In the brain, OC significantly reduced Aβ_40_ by 40% and 73% in 4- and 9-month-old mice, respectively, and Aβ_42_ levels by 45% in 9-month-old 5xFAD mice. This effect was associated with a noticeable significant reduction in IgG extravasation (Figure 2), which is consistent with our previous results [27,28].

## 3. Discussion

Aging and AD alter body metabolism [30,31], which could be modulated by lifestyle changes and therapeutic treatments. The objectives of this work were to (1) assess changes in metabolic and behavior phenotypic parameters with age and AD pathology in WT and 5xFAD mice; (2) associate sleep disturbances with BBB dysfunction and Aβ brain levels; and (3) evaluate the effect of pathology modulation by OC treatment on the metabolic and behavior parameters. Our findings demonstrate metabolic and behavioral alterations in WT and 5xFAD mouse models with age and pathology. The findings also revealed a correlation between BBB leakage and brain soluble Aβ levels and, for the first time, a correlation between BBB leakage and sleep time. In addition, 5xFAD mice treatment with OC modulated metabolic and behavioral parameters, with a more prominent effect in the older 5xFAD mice.

AD patients have lower body weight than cognitively normal individuals of the same age due to appetite and metabolic state changes [6,32]. In addition, dehydration is one of the symptoms induced in AD patients [33]. In our studies in mice, we observed that 5xFAD-9m mice have lower body weight than WT-9m, although food intake was the same in both strains. The reduced body weight at an older age could be related to the general increase in body metabolism represented by the increased dehydration rate (parameter VH_2_O), hyperventilation (parameters VO_2_, VCO_2_, and RER), activity rate (parameter EE), movement (traveled distance), and sleep disturbances, all of which could be associated with anxiety-like behavior in mice.

With normal aging in WT mice, we observed a decreased activity, metabolism, and movement, which is in contrast to the effect in 5xFAD mice where a higher activity and metabolism with greater movement was determined, suggesting Aβ and related pathology in 5xFAD mice is contributing to the observed effect, thus to the anxiety-like behavior. Previous assessments of anxiety-like behavior in mouse models of AD demonstrated inconsistent and contradictory results. For example, in some studies, 5xFAD and APP/PS1 mice exhibited decreased or equivalent anxiety-like behavior in the open field or elevated plus maze relative to WT mice [34,35,36]. On the other hand, others have reported increased anxiety-like behavior in 5xFAD mice and other AD mouse models [37,38,39]. Such inconsistencies in the results could be related to the experimental conditions. For example, the elevated plus-maze tests are frequently used to assess mouse anxiety by locating them at a specific height, which introduces a fear factor by exposing them to the open and height, while the phenotypic behavior monitored in this work is fear-free. The mouse anxiety assessment was performed in the home cage under standard housing conditions, which is more similar to that in humans. Indeed, additional studies are necessary to correlate the anxiety-like behavior determined from the metabolic and behavior parameters with those determined using open field and elevated plus-maze tests under the same experimental conditions.

Furthermore, with aging, the elderly experience a sleep–wake disruption induced by physiological changes, such as aging, or due to the presence of a disease condition [40]. The elderly tend to have more frequency of taking light sleep than deep sleep, suggesting a less efficient circadian behavior [40]. Sleep disturbance is considered one of the well-known behavioral phenotypes of AD [41], which worsens as the disease progresses [8,42]. The progressive neuropathological alteration and Aβ burden in AD have been associated with sleep dysregulation, thus impacting the sleep–wake activity [42,43,44]. When tested in WT mice, the cerebral injection of Aβ_25–35_ significantly reduced non-rapid eye movement sleep and increased wakefulness [45,46], suggesting a role for Aβ in sleep disturbances. With aging, we observed that the 9-month-old WT mice have reduced sleep time compared to the 4-month-old young mice. These findings are consistent with those reported by Soltani and colleagues, who assessed the effect of aging on the sleep–wake cycle and concluded that the induced sleep disorder in 12-month-old C57BL/6 mice was comparable to that in 3-month-old mice [47]. In the AD mouse model, 5xFAD mice also exhibited a significantly reduced sleeping time at both ages compared to the WT mice supporting that in AD, besides aging, the sleep pattern is influenced by Aβ pathology.

To correlate changes in metabolic and behavioral phenotypes with Aβ and related pathology as a function of age and pathology, we assessed plasma and brain Aβ levels and the BBB function. As the disease progressed with age, the accumulation of Aβ increased in the 5xFAD mouse brains. However, in plasma, while the low Aβ levels were not significantly altered in the WT mice with age, in 5xFAD mice, the plasma levels of Aβ were significantly higher in 4-month-old compared to 9-month-old mice. Neurotoxic agents, such as Aβ, are cleared, at least in part, from the brain to the blood across the BBB [48]. At 4 months of age, the higher plasma levels of Aβ in 5xFAD than WT could be due to the increased production of brain Aβ that gets cleared to the blood across the BBB [49,50]. In contrast, the reduced Aβ plasma levels in 5xFAD-9m mice relative to 5xFAD 4-m and WT-9m mice could be explained, at least in part, by reduced Aβ clearance across the BBB and its brain accumulation. In AD, as the disease progresses, the elimination of Aβ is reduced due to reduced degradation and clearance across the BBB, leading to brain Aβ accumulation and plaque formation [49,50,51]. In AD mouse models, increased brain Aβ is associated with BBB breakdown supported by the increased IgG extravasation, which significantly increased as the disease progressed. Our correlation studies showed a positive correlation between brain Aβ and IgG extravasation and a negative correlation between total sleep hours and IgG extravasation, which suggests the association of BBB breakdown with reduced sleep time, an effect that is mediated by Aβ; however, the correlation between soluble Aβ and sleep hours was not strong. Indeed, additional studies are required to explain these findings.

To assess the effect of AD treatment on the metabolic and behavior parameters and whether they can be rectified, we used the phenolic compound oleocanthal (OC) as a model molecule. We previously reported that OC at 5 and 10 mg/kg doses demonstrates a protective and therapeutic effect against Aβ and related pathology in AD mouse models [25,26,27,28]. In addition, we also reported that OC crosses the BBB, where we detected it in mouse brains following an intravenous administration [52]. In the current study, 5xFAD mice treatment with 10 mg/kg OC modulated the metabolic and behavior parameters to values approaching the WT mice, with the effect being more pronounced in the 9-month-old than the 4-month-old 5xFAD mice. OC treatment increased the body weight of mice, which could be explained, at least in part, by the reduced activity rate and anxiety-like behavior. OC also improved sleeping time during the day to values comparable to the WT mice of the same age suggesting its beneficial effect against anxiety and sleep disturbances. This observed effect with OC was associated with reduced brain Aβ levels and improved BBB function. The effect of OC on plasma Aβ levels differed between 5xFAD-4m (i.e., early disease stage) and 5xFAD-9m (i.e., with advanced pathology), where OC reduced plasma Aβ in the former and increased it in the latter. Indeed, additional studies are necessary to explain these results; however, we speculate that the dysregulated balance between Aβ production and clearance, and the difference in this dysregulation between early and advanced pathology influenced the effect of OC. For example, vehicle-treated 5xFAD-4m mice demonstrated higher plasma Aβ levels than 5xFAD-9m mice, suggesting the better clearance of produced soluble Aβ to the blood across the BBB. However, as the pathology advances, at 9 months and due to increased aggregation and deposition of produced Aβ in the form of plaques, the reduced available soluble Aβ for clearance across the dysfunctional BBB could impact Aβ plasma levels, which collectively were rectified by OC treatment. We previously reported that OC reduces brain Aβ production and increases its clearance across the BBB [26,50,53], which, thus, could explain the observed effect.

This study has several limitations, notably the use of male WT and 5xFAD mice and not including female mice, which require evaluation. Besides, additional studies using conventional tasks such as the open field test could be necessary to confirm the anxiety-like behavior and its reduction by OC treatment, which are planned for future investigation. Furthermore, additional studies are required to confirm and explain our correlation studies, including the correlation between sleep and soluble Aβ, as well as with total Aβ (soluble and insoluble).

In conclusion, we observed metabolic and behavioral alterations with age and pathology in WT and 5xFAD mouse models. With aging, 5xFAD mice demonstrate a reduced body weight, increased metabolic activity rate, and increased anxiety-like behavior, opposite to those observed in WT mice. In addition, both mouse models demonstrated reduced sleep hours, with 5xFAD mice showing less sleeping time than WT mice. Our findings also reveal a relationship between reduced sleep duration and BBB breakdown for the first time. Furthermore, 5xFAD mice treatment with OC ameliorated the assessed metabolic and behavior parameters. OC improved anxiety-like behavior symptoms and increased sleeping hours, two major symptoms of AD. In conclusion, our findings indicate that the metabolic and behavioral parameters assessed in this study could be used as assessment tools for disease progression, severity, and treatment efficacy in mouse models of AD.

## 4. Materials and Methods

### 4.1. Animals

Male wild-type C57BL/6J (Strain #:000664) and 5xFAD (Strain #: 034848-JAX; both from Jackson Laboratory, Bar Harbor, ME, USA) mouse models were used in the studies at 4 and 9 months of age (*n* = 10 mice per group). In addition to these four groups, other two groups were added (*n* = 10 mice/group), namely, 5xFAD-4-month-old and 5xFAD-9-month-old that received 10 mg/kg OC by oral gavage daily for 3 months starting at the age of 1 and 6 months, respectively. WT and 5xFAD mice received saline (as a vehicle) by oral gavage. OC is an amphiphilic compound and is water soluble [54]. A schematic diagram of the experimental design is demonstrated in Scheme 1. The AD mouse model 5xFAD expresses human amyloid precursor protein (APP) with the mutations APP KM670/671NL (Swedish), APP I716V (Florida), APP V717I (London), and PSEN1 M146L and PSEN1 L286V, leading to early and aggressive Aβ accumulation associated with deficits in spatial learning as the disease progresses [55]. In 5xFAD mice, extracellular Aβ plaque deposition starts at 2 months with gliosis. This early Aβ deposition and gliosis induce synaptic loss, resulting in cognitive impairment at 4 months. Aged C57BL/6J mice are frequently used in studies related to neurodegenerative disorders. C57BL/6 mice demonstrate a decline in physical function as early as 6 months of age, while the cognitive function begins to decline later, with a considerable impairment present at 22 months of age [56]. WT and 5xFAD mice were housed for breeding in plastic containers under 12 h light/dark cycle, 22 °C, 35% relative humidity, and *ad libitum* access to water and food. At 4 and 9 months of age, mice were transferred to metabolic cages as described below to perform the metabolic and behavioral phenotypes assessments. All animal experiments and procedures were approved by the Institutional Animal Care and Use Committee of Auburn University and according to the National Institutes of Health Guidelines Principles of Laboratory Animal Care.

### 4.2. Metabolic and Behavioral Phenotyping Assessments

Promethion metabolic mouse cages (Sable Systems, Las Vegas, NV, USA) were used to house animals for metabolic screening and phenotyping. Animals were transferred from their home cages and singly housed in the metabolic cages at 4 and 9 months of age. The animals were housed in the metabolic cages for 36 h, with the first 12 h stated for the cage environment adaptation and the next 24 h for data collection. Animal activity was measured using Promethion XYZ Beambreak Activity Monitor. Food and water intake, body weight, movement distance, sleeping time, VO_2_, and VCO_2_ were measured using Promethion precision MM-1 Load Cell Sensors. The time for metabolic parameters measurement is defined as 12h:12h light:dark cycle with ZT 0 (representing lights on) and ZT12 (representing lights off). The amount of food and water withdrawn from the container was measured and analyzed. The body weight monitors were plastic tubes that also function as in-cage enrichment and nesting devices. VH_2_O, VCO_2_, and VO_2_ (all measured in mL/min) were analyzed using the Promethion GA-3 gas analyzer to provide detailed respirometry data. Mean energy expenditure (EE) was calculated in kilocalories/hour (kcal/h) by utilizing the Weir equation: 60 × (0.003941 × VO_2_ (n) + 0.001106 × VCO_2_ (n)). ANCOVA was used to adjust for the influence of body weight as a covariate on EE and VO_2_ using custom R scripts based on the multiple linear regression analysis described on the MMPC Energy Expenditure analysis page [57]. RER was determined by measuring gas exchange within the metabolic cages to identify the substrate primarily utilized for energy within the body. Specifically, RER is the ratio of VCO_2_ produced to VO_2_ (RER = VCO_2_/VO_2_). All metabolic phenotyping data were analyzed using ExpeData software (version 1.8.2; Sable Systems) with Universal Macro Collection (version 10.1.3; Sable Systems). The parameter distance traveled is the sum of all distances traveled within the beam break system in meters (m), including fine movement (such as grooming and scratching) and direct locomotion.

### 4.3. Immunofluorescence Staining

After the phenotyping assessment, mice were sacrificed to collect the blood and brain tissues. Brain sections of 15 μm were prepared using a ThermoScientific HM525 NX Cryostat (Waltham, MA, USA). Sections were fixed with 4% paraformaldehyde and then blocked with the blocking buffer TrueBlack background suppressor (Biotum; Fremont, CA, USA) for 60 min. IgG extravasation from brain microvessels was determined to assess BBB integrity. For this, sections were probed using dual immunohistochemical staining with anti-rabbit collagen-IV as the primary antibody to detect brain microvessels (Millipore Sigma, Burlington, MA, USA) and Alexa Fluor^®^ 488-conjugated goat anti-mouse IgG H&L (Abcam, Cambridge, UK) to detect IgG extravasation, both at 1:500 dilution. The secondary antibody for collagen-IV antibody was anti-rabbit (Alexa Fluor^®^ 594) (Abcam). For each treatment, image acquisition was performed in 10 tissue sections spanning the hippocampus and cortex, each separated by 150 μm (total of 20 sections per mouse). Images were captured and adjusted to the lowest background signal using Nikon Eclipse Ti-S inverted fluorescence microscope (Melville, NY, USA). To quantify IgG extravasation, sections were normalized to the same background. Images were analyzed using Image J software (National Institutes of Health, Bethesda, MD, USA) that was set for mean value, minimum value, maximum value, and limit of the threshold followed by analysis.

### 4.4. Measurements of Brain and Plasma Aβ Using ELISA

Commercially available ELISA kits were used to determine Aβ_40_ and Aβ_42_ levels in WT and 5xFAD mice brain tissue lysates and plasma according to the manufacturer’s instructions (R&D Systems, Minneapolis, MN, USA). All samples were run in duplicate. Brain Aβ levels were corrected to the total protein amount in each sample using the bicinchoninic acid (BCA) assay.

### 4.5. Statistical Analysis

All metabolic phenotype parameters were analyzed with RStudio and the Rx64 3.6.0 software environment (RStudio, PBC, Boston, MA, USA). Twenty-four-hour circadian data were analyzed using the random-effects model to account for the repeated measures from an individual animal. A one-way ANOVA test with *Tucky post hoc* using GraphPad Prism (San Diego, CA, USA) was used to evaluate the difference between the three groups. Student’s *t*-test was used to evaluate differences between the two groups. The nonparametric Spearman correlation with a two-tailed *p*-value is used for the correlation analysis. Significance for all measures was determined at *p* < 0.05, and all data are presented as mean ± SEM.

## Figures and Tables

**Table 1:** A summary of the assessed metabolic parameters with their abbreviations.

Parameters	Definition
Body weight	Mean body mass of the animal, in grams (g)
Food intake	Mass of food consumed by animal, in gram (g)
Water intake	Mass of water consumed by animal, in gram (g)
EE	Mean energy expenditure, in kcal/h
VCO_2_	Mean rate of carbon dioxide emission, in mL/min
VO_2_	Mean rate of oxygen consumption in mL/min
RER	Respiratory exchange ratio, VCO_2_/VO_2_, unit-less
VH_2_O	Mean rate of water vapor loss, in mL/min
Distance travelled	Sum of all distances traveled, in meters
Cumulative distance	Sum of cultivating distance traveled, in meters
Sleep	Sleep time (h) = quiet for > 40 s

## Data Availability

The data supporting this paper and other study findings are available from the corresponding author upon reasonable request.

## Supplementary Materials

The following supporting information can be downloaded at: https://www.mdpi.com/article/10.3390/molecules28145592/s1, Figure S1: The effect of aging on changes in metabolic parameters in 4- vs. 9-month-old WT mice with time for 24 h. Data are presented as mean ± SEM for n = 10 mice/group for each time point; Table S1: Statistical significance of WT-4 vs. WT-9 months shown in Figure S1; Figure S2: The effect of aging on changes in metabolic parameters in 4- vs. 9-month-old 5xFAD mice with time for 24 h. Data are presented as mean ± SEM for n = 10 mice/group for each time point; Table S2: Statistical significance of 5xFAD-4 vs. 5xFAD-9 months shown in Figure S2; Figure S3: The effect of pathology on changes in metabolic parameters in 4-months old WT vs. 5xFAD mice with time for 24 h. Data are presented as mean ± SEM for n = 10 mice/group for each time point; Table S3: Statistical significance of 4 months WT vs. 5XFAD in Figure S3; Figure S4: The effect of pathology on changes in metabolic parameters in 9-months old WT vs. 5xFAD mice with time for 24 h. Data are presented as mean ± SEM for n = 10 mice/group for each time point; Table S4: Statistical significance of 9 months WT vs. 5xFAD in Figure S4.
