# Supplementary material for: Oleocanthal Ameliorates Metabolic and Behavioral Phenotypes in a Mouse Model of Alzheimer’s Disease
Source: Molecules. 2023 Jul 23;28(14):5592. doi: 10.3390/molecules28145592 (PMC10385639; doi:10.3390/molecules28145592)
Supplement: Supplementary file 1 [file molecules-28-05592-s001.zip › molecules-2474955-supplementary.pdf]

Supplementary data

Figure S1. The effect of aging on changes in metabolic parameters in 4- vs. 9-month-old WT mice with time for 24 h. Data are presented as mean + SEM for n = 10 mice/group for each time point.

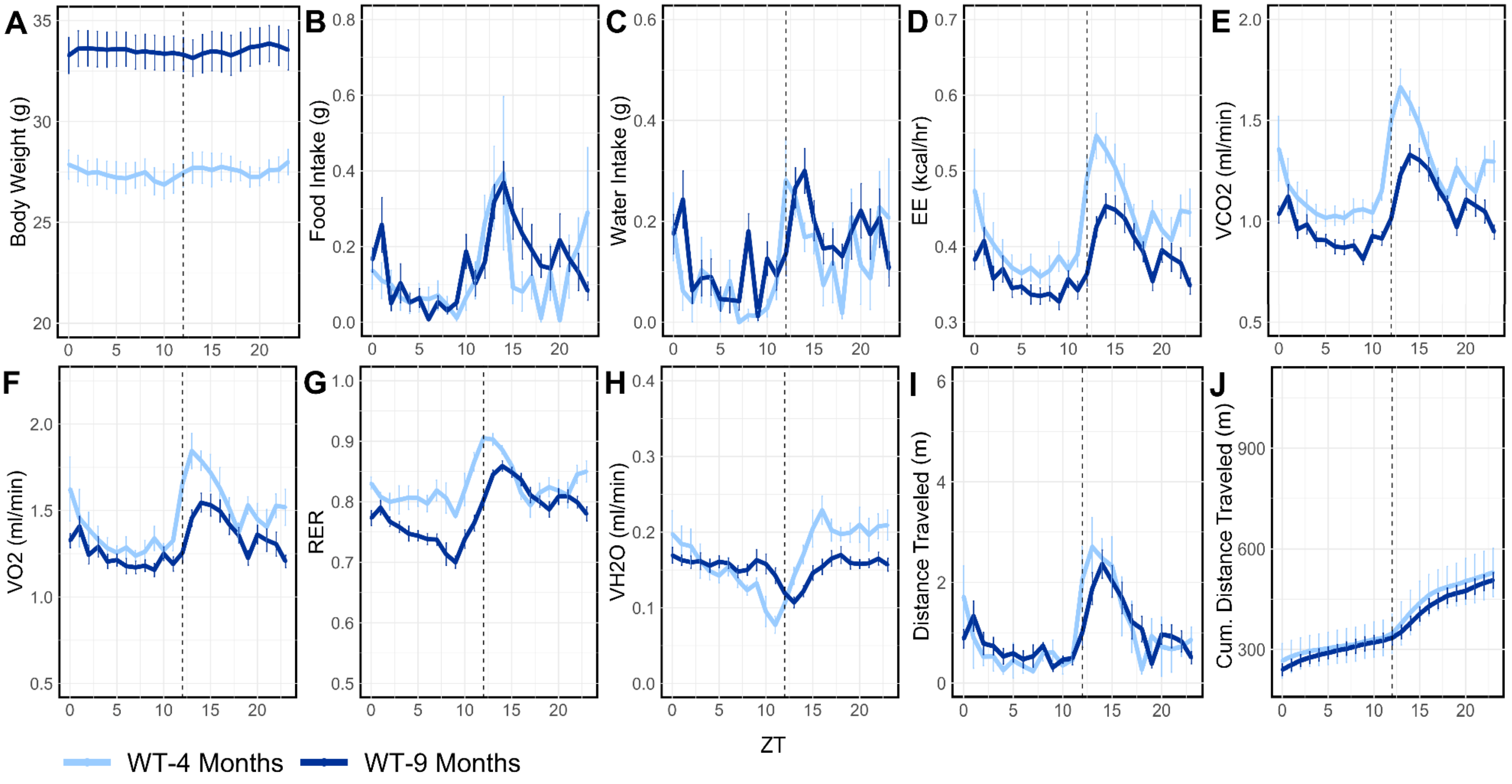

Table S1. Statistical significance of WT-4 vs. WT-9 months shown in Figure S1.

ZT	0	1	2	3	4	5	6	7	8	9	10	11	12	13	14	15	16	17	18	19	20	21	22	23
Body Weight	*	*	*	*	*	*	*	*	*	*	**	*	*	*	*	*	*	*	*	*	**	*	*	*
Food Intake																								
Water Intake																								
EE	*												**	**						*				*
VCO2	*												***	**						*				*
VO2													**	**						*				*
RER								**	**	*	**	***	***											*
VH2O											*	*				*	**							
Traveled distance																								
Cumulative traveled distance																								

$p<0.05$ =*, $p<0.01$=**, $p<0.001$=***

Figure S2. The effect of aging on changes in metabolic parameters in 4- vs. 9-month-old 5xFAD mice with time for 24 h. Data are presented as mean + SEM for n = 10 mice/group for each time point.

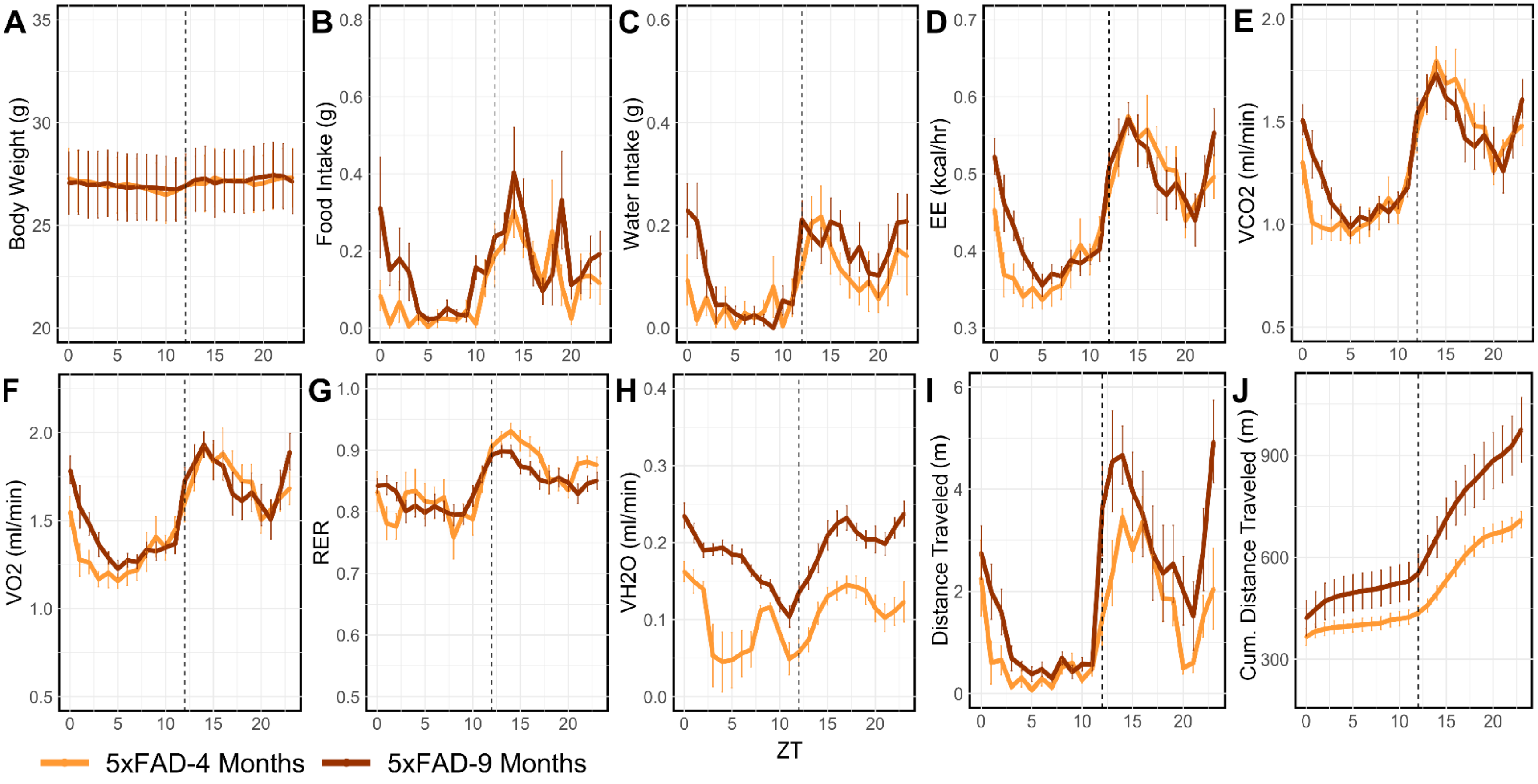

Table S2. Statistical significance of 5xFAD-4 vs. 5xFAD-9 months shown in Figure S2.

ZT	0	1	2	3	4	5	6	7	8	9	10	11	12	13	14	15	16	17	18	19	20	21	22	23
Body Weight																								
Food Intake																								
Water Intake		*																						
EE		*																						
VCO2		*																						
VO2																								
RER																								
VH2O	**	*		***	***	***	***	***					**	**	**	**	**	**	**	*	***	***	***	***
Traveled distance													**	**										***
Cumulative traveled distance																*	*	*	*	*	*	**	**	**

p<0.05 =*, p<0.01=**, p<0.001=***

Figure S3. The effect of pathology on changes in metabolic parameters in 4-months old WT vs. 5xFAD mice with time for 24 h. Data are presented as mean + SEM for n = 10 mice/group for each time point.

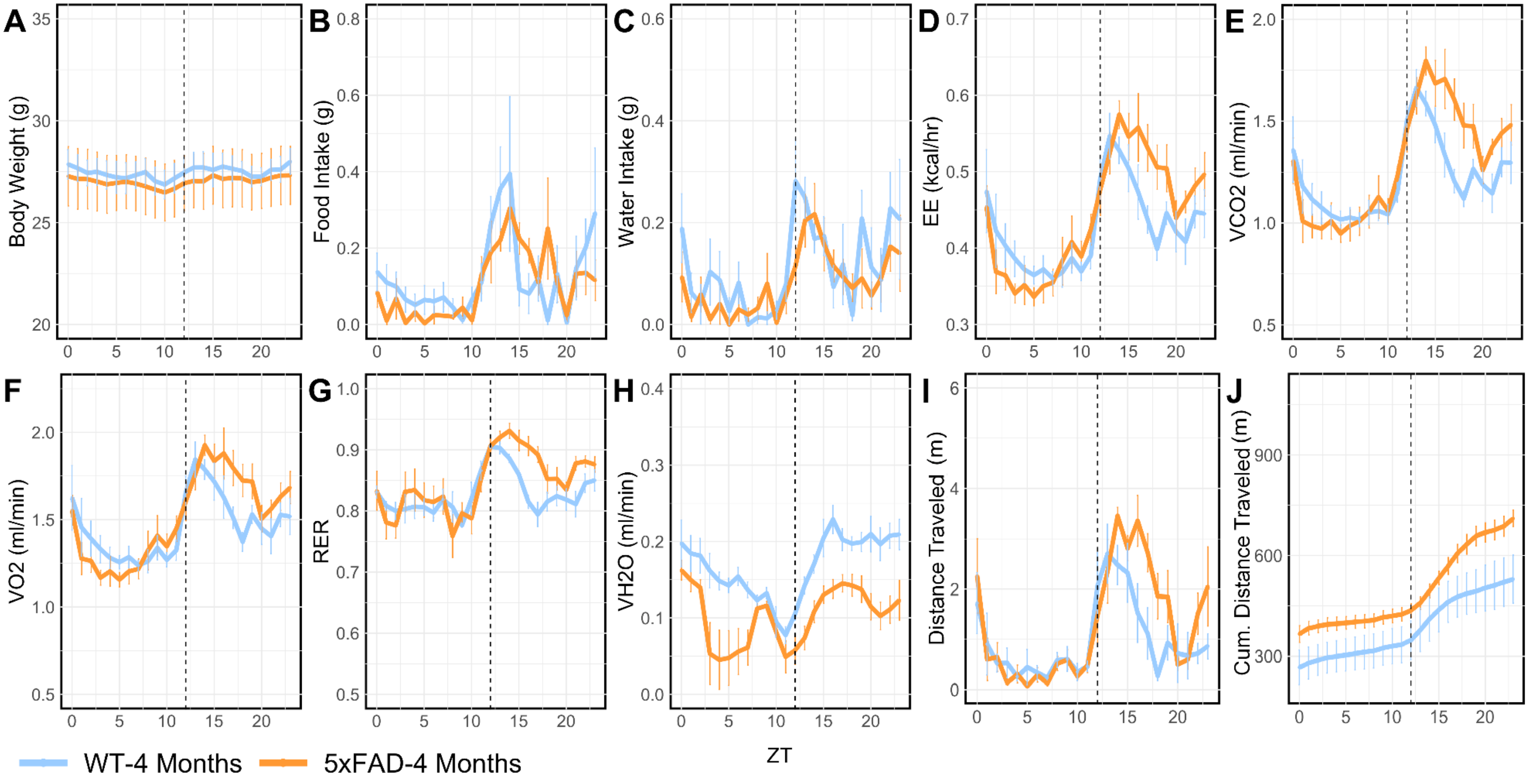

Table S3. Statistical significance of 4 months WT vs. 5XFAD in Figure S3.

ZT	0	1	2	3	4	5	6	7	8	9	10	11	12	13	14	15	16	17	18	19	20	21	22	23
Body Weight																								
Food Intake																								
Water Intake																								
EE																			*					
VCO2																	*	*	*					
VO2																								
RER																	*	**						
VH2O				***	***	**	**	*						*		*	**				**	**	**	**
Traveled distance																								
Cumulative traveled distance																								

$p<0.05$ =*, $p<0.01$=**, $p<0.001$=***

Figure S4. The effect of pathology on changes in metabolic parameters in 9-months old WT vs. 5xFAD mice with time for 24 h. Data are presented as mean ± SEM for n = 10 mice/group for each time point.

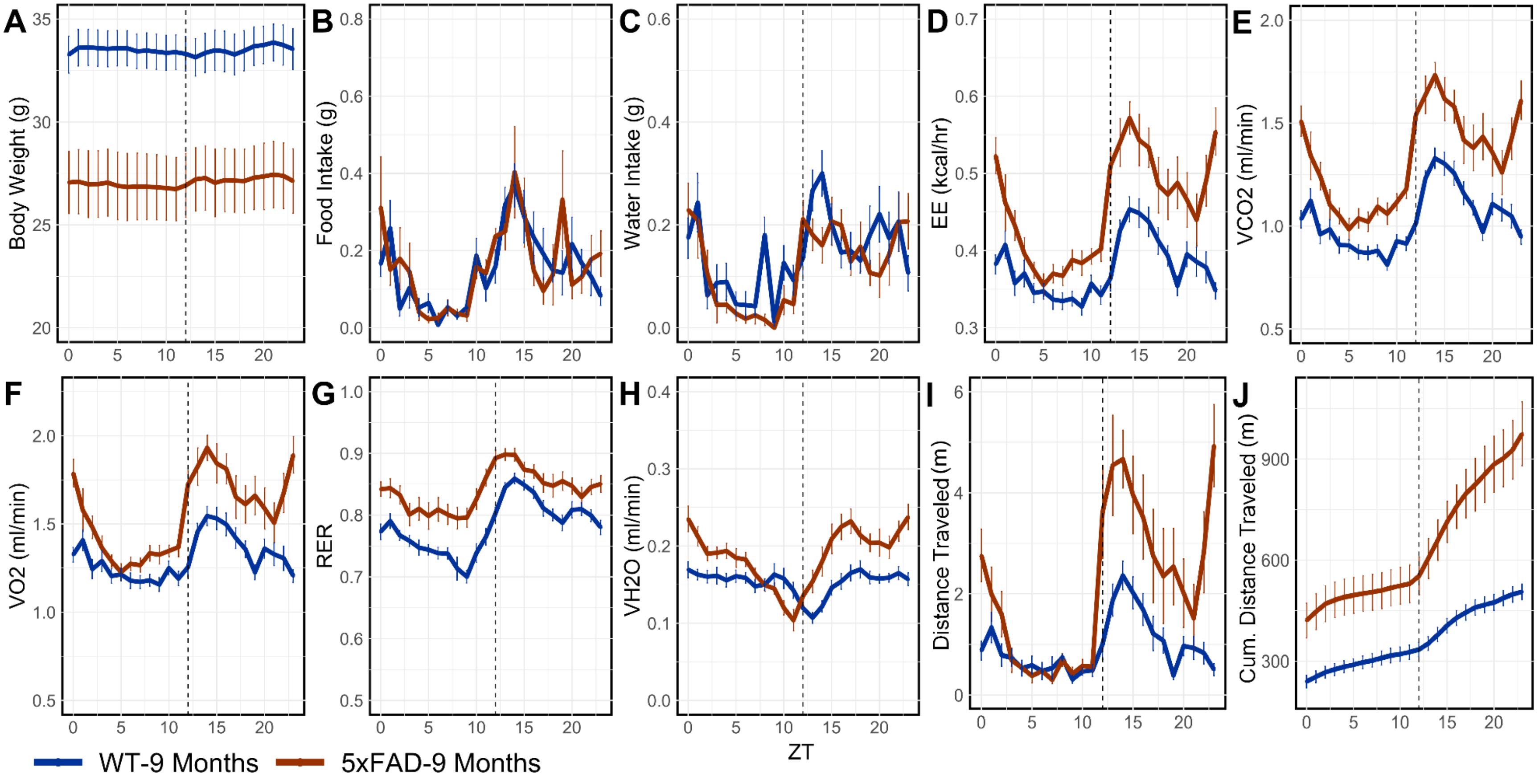

Table S4. Statistical significance of 9 months WT vs. 5xFAD in Figure S4.

ZT	0	1	2	3	4	5	6	7	8	9	10	11	12	13	14	15	16	17	18	19	20	21	22	23
Body Weight	**	**	***	***	**	***	***	**	***	**	**	***	**	**	**	**	**	**	**	**	**	***	**	**
Food Intake																								
Water Intake									*															
EE	***		*										***	***	***	**	**		*	***			**	***
VCO2	***		*							*		*	***	***	***	**	**	*	**	***	*		***	***
VO2	***												***	**	***	**	**		*	***			**	***
RER	**	*	**		*	*	**	*	***	***	***	***	***	*						**				**
VH2O	**	*												*	**	**	***	**			*		*	***
Traveled distance	**												***	***	***	**	**	*		***			**	***
Cumulative traveled distance	**	**	**	**	**	**	**	**	**	**	**	**	**	***	***	***	***	***	***	***	***	***	***	***

p<0.05 =*, p<0.01=**, p<0.001=***
